# Acknowledgment of Invited Editors

**DOI:** 10.1128/mBio.00414-20

**Published:** 2020-03-24

**Authors:** Arturo Casadevall

**Affiliations:** a Johns Hopkins University School of Medicine, Baltimore, Maryland, USA

## EDITORIAL

On behalf of the editors of *mBio*, I gratefully acknowledge the following individuals, who served as invited editors for the journal in 2019. While not members of the Board of Editors, invited editors serve an important role in the review process. Invited editors are experts in their fields of research who add an additional level of quality to the review process. An editor may assign a paper to an invited editor when he/she would like to have an additional expert opinion of the reviews or when the subject area falls outside the editor’s primary area of expertise.

The time and effort of the following experts in handling articles have been essential to ensuring the high quality of our publications, and their help is greatly appreciated.

Christopher Aiken
Brian J. Akerley
Kevin Alby
Irving Coy Allen
M. Javad Aman
Gaya Amarasinghe
Richard Frederick Ambinder
Karthik Anantharaman
John M. Archibald
A. Elizabeth Arnold
Marc Bailly-Bechet
Ralph S. Baric
Harris D. Bernstein
Lionel Berthoux
Sonja M. Best
Fabiana Bigi
David F. Blair
Daniel R. Bond
Robert A. Britton
Shilpa Buch
Daniel H. Buckley
Karen Bush
Vern B. Carruthers
Thomas A. Clarke
Anthony J. Clarke
Janice Clements
John M. Coffin
Vaughn S. Cooper
Laura M. Cox
Keith A. Crandall
Brian Crane
Alison K. Criss
Bryan R. Cullen
Bryan W. Davies
Denise Dearing
Joseph P. Dillard
Daniel DiMaio
Victor J. DiRita
Daniel Distel
Philipp Engel
Slava Epstein
Tobias J. Erb
Edward J. Feil
Paul D. Fey
Alain Filloux
Suzanne M. J. Fleiszig
Erik K. Flemington
Samuel C. Forster
Jarrod R. Fortwendel
Kevin Foster
Eric O. Freed
Attila Gacser
Shou-Jiang Gao
Jean-Marc Ghigo
Zemer Gitai
Benjamin Glick
Erin D. Goley
Sabina Gorska
Heinrich Georg Gottlinger
Shipra Grover
Nathan Grubaught
Angelika Gründling
John Guatelli
Thomas Guillard
Maude Guillier
Albert Haas
Maria Hadjifrangiskou
Susan Hafenstein
Stephen L. Hajduk
Christopher Hayes
Robert A. Heinzen
Andrew J. Henderson
Tory A. Hendry
Gerhard J. Herndl
Mark C. Herzberg
Hubert Hilbi
J. Robert Hogg
Alexander R. Horswill
Marcus A. Horwitz
Benjamin A. Horwitz
Diarmaid Hughes
Kelly T. Hughes
Kevin Hybiske
Nicole Hynson
Tetsuro Ikegami
James Imlay
Jean-Luc Imler
Ralph R. Isberg
Mary Jackson
Urs Jenal
Jonathan J. Juliano
Suckjoon Jun
Ganjam V. Kalpana
Barbara I. Kazmierczak
Lance E. Keller
Patricia J. Kiley
Keith P. Klugman
Gary P. Kobinger
Eugene V. Koonin
Richard A. Koup
Gordon Langsley
Michael T. Laub
Benhur Lee
Salome LeibundGut-Landmann
Jose Lemos
Stuart M. Levitz
Kim Lewis
Amanda L. Lewis
Melody M. H. Li
Mathias Lichterfeld
Gerard Lina
Per O. Ljungdahl
Erwin London
Carole A. Long
Andrew J. Macpherson
Magdalene So
Esperanza Martínez-Romero
Laura-Isobel McCall
John McDowell
Alan McNally
Monica Medina
Peter Mergaert
Álvaro San Millan
Olivo Miotto
Volker Mueller
Timothy F. Murphy
William W. Navarre
Stuart J. Neil
Jerome Nigou
Juan Nogales
Mairi C. Noverr
Anil Ojha
Beth Orcutt
Eric Oswald
George O’Toole
Kelli L. Palmer
Tracy Palmer
John S. Parker
Kabir Peay
Steve Perlman
Mariya Ivanova Petrova
Liise-anne Pirofski
Mariagrazia Pizza
Massimo Pizzato
Paul J. Planet
Richard K. Plemper
Spyros Pournaras
Michelle Lynne Reniere
Federico Rey
Eduardo P. C. Rocha
Antonis Rokas
Jason W. Rosch
Oren Rosenberg
Manish Sagar
Jason W. Sahl
Dominique Sanglard
Alyson Santoro
Reetta Satokari
Larry S. Schlesinger
Thomas Mitchell Schmidt
Michael J. Schurr
Joshua Shaevitz
Donald C. Sheppard
David Sibley
Robert F. Siliciano
Guido Silvestri
Jorg Soppa
Alfred Michael Spormann
Stephen Stearns
William Sugden
Ronald Swanstrom
David G. Thanassi
Thomas C. G. Bosch
Leann Tilley
Michal Toborek
Sue A. Tolin
Cagla Tukel
Akhil B. Vaidya
Raphael Valdivia
Susana T. Valente
Kendra Vehik
Warren Wakarchuk
Yue Wang
Scott C. Weaver
Sean P. J. Whelan
Katrine L. Whiteson
Chris Whitfield
Richard J. Whitley
Gary R. Whittaker
K. Eric Wommack
Daniel J. Wozniak
Fitnat H. Yildiz
Ozlem Yilmaz
Paul R. Young
Egija Zaura
Liping Zhao

